# Bread Wheat Landraces Adaptability to Low-Input Agriculture

**DOI:** 10.3390/plants12132561

**Published:** 2023-07-06

**Authors:** Evangelos Korpetis, Elissavet Ninou, Ioannis Mylonas, Georgia Ouzounidou, Ioannis N. Xynias, Athanasios G. Mavromatis

**Affiliations:** 1 Institute of Plant Breeding and Genetic Resources, Hellenic Agricultural Organization DIMITRA, 57001 Thessaloniki, Greece; ekorpetis@elgo.gr; 2 Department of Agriculture, International Hellenic University, Sindos, 57400 Thessaloniki, Greece; lisaninou@agr.teithe.gr; 3 Institute of Technology of Agricultural Products, Hellenic Agricultural Organization DIMITRA, S. Venizelou 1, Lycovrissi, 141 23 Attika, Greece; geouz@nagref.gr; 4 School of Agricultural Technol. & Food Technol. and Nutrition, University of Western Macedonia, 53100 Florina, Greece; ixynias@teiwm.gr; 5 Laboratory of Genetics and Plant Breeding, School of Agriculture, Aristotle University of Thessaloniki, 54124 Thessaloniki, Greece; amavromat@agro.auth.gr

**Keywords:** bread wheat, Greek landraces, field evaluation, low inputs, GGE biplot

## Abstract

Bread wheat landraces were an important source of biodiversity used in agriculture before the widespread adoption of high-yielding commercial cultivars adapted to high inputs. Could future agriculture exploit these landraces in different cropping systems in organic or lower-input environments? A two-year field trial was conducted to evaluate grain yield, agronomic performance, and grain quality of bread wheat landraces under different cropping systems, including low-input/organic/conventional environments. Significant variability was found for almost all characteristics among landraces, which makes landraces valuable sources of genetic variation for breeding programs aimed at achieving high and consistent production as well as high-quality products in low-input/organic environments. Additionally, landraces play a crucial role in expanding the genetic diversity of cultivated bread wheat and mitigating biodiversity erosion, thereby enabling crops to better withstand the challenges of low-input/organic agriculture. The landrace “Xilokastro Lamias” had the highest yield among the landraces evaluated in the first growing season (2.65 t·ha^−1^) and one of the highest yields (2.52 t·ha^−1^) of all genotypes in the second growing season, which shows promising potential as a starting material in breeding programs targeting high and stable yields. GGE biplot analysis identified the landrace ”Xilokastro Lamias”, along with commercial cultivars “Yecora E” and “Panifor”, as suitable candidates for direct use in low-input/organic wheat farming systems to achieve enhanced productivity. In the conventional environment (C2-IPGRB), commercial cultivars showed the highest values (3.09 to 3.41 ton·ha^−1^). Of the landraces, only the X4 showed a high GY (3.10 ton·ha^−1^) while the other landraces had ~33–85% lower yield. In the organic environment (O2-IPGRB), the highest productivity was found in the commercial cultivar X5 and the landrace X4. Commercial cultivars X8 and X7 showed ~68% reduction in GY in the organic environment compared to the conventional, while this reduction was half for the landraces. Finally, the reduction in grain yield between conventional and organic environments was observed to be 45% for commercial cultivars, while it was only half for landraces. This finding confirms the adaptability of landraces to organic agriculture.

## 1. Introduction

Wheat is one of the most important staple crops, which, in the last decade, has been cultivated worldwide on about 218.5 million hectares with an average annual production of 740 million tons and an average annual grain yield of 3.4 tons per hectare [1]. About 95% of world wheat production is bread wheat, while the remaining 5% is covered by durum wheat [2,3], which is of special interest in the Mediterranean basin [4,5]. Most of the wheat-growing areas are in the northern hemisphere of the earth. The distribution of wheat in them is related to the various species and varieties as well as their adaptability to the various environments [6,7]. In Greece in recent years, wheat has occupied approximately 17.5% of the total cultivated area, with bread wheat reaching an average of 1.5 million hectares and durum wheat at 4.5 million hectares [8].

In the last century, the improvement of wheat has turned to the development of cultivars with high and stable yields, better product quality, and resistance to biotic and abiotic stresses. The achievement of these goals came through the Green Revolution and the introduction of dwarf genes into modern semi-dwarf wheat cultivars that are adapted to high-input agriculture (synthetic chemical fertilizers, fungicides, pesticides). These new elite wheat cultivars came from a narrow germplasm pool [9], as breeders are often afraid of losing the co-adapted gene complexes and the linkage drag, and they will need more time to develop new cultivars if they use other exotic germplasm [10].

According to Villa et al. [11], landraces are defined as dynamic populations with the historical origin, distinct identity, and high variability, locally adapted and related to traditional cultivation systems, while according to Dwivedi et al. [10] respond to current and emerging challenges for agriculture in stress environments. Wheat landraces have a high tolerance to biotic and abiotic stresses, high yield stability, and average yield in low-input environments [4,12,13,14] and have not undergone conscious selection by man [15,16].

Wheat landraces may have some advantages over commercial cultivars in organic and low-input environments because they have been selected over time for their ability to adapt to local growing conditions and resist pests and diseases without the use of synthetic fertilizers, pesticides, or other inputs. Commercial wheat cultivars, on the other hand, have been developed for high yields and uniformity under high-input conditions and may not be suitable for organic or low-input environments. These high-input cultivars often require more fertilizers, pesticides, and other inputs to achieve their full potential and may be more susceptible to pests and diseases in the absence of these inputs.

The gradual replacement of landraces by new, improved varieties has led to the removal from the cultivation of part of the traditional genetic material, contributing to the erosion of biodiversity [14,17]. The superiority of the modern improved varieties over the landraces is due to earliness, dwarfism, and resistance to lodging.

The narrow genetic composition of modern cultivars affects their ability to adapt. So, they are susceptible to stresses occurring in their environment. Landraces show heterogeneity and low yield in grain but show stable performance in a wide range of environments, resulting in greater stress tolerance [18].

The genomes of modern wheat cultivars can be considered as mosaics derived from the genomes of landraces, which were selected as starting genetic material for systematic wheat breeding at the beginning of the 20th century [19,20]. Much research has focused on the benefits of genes and alleles to interpret the genotypic effects of selection for different environments [21]. Exploiting these results can lead to the introduction of QTL/genes/alleles from landraces to modern wheat cultivars using biotechnology tools, thus improving the potential of modern cultivars for better growth, adaptation, and productivity in environments strongly affected by the current climate changes.

The lack of modern wheat cultivars adapted to reduced inputs or organic farming systems has highlighted the importance of preserving and using landraces, which are generally adapted to such farming systems [22]. However, to further optimize the quality and yield stability of organic products, new cultivars that are adapted to organic farming systems are required. The desirable characteristics of the cultivars include adaptation to organic management of soil fertility, which means low and organic inputs, a better root system, and the ability to interact with beneficial soil microorganisms and suppress weeds, contributing to the improvement of soil, health of crops and seeds, good quality of the products, and high level and stability of yield. However, to date, many of the desired features have not received the necessary priority in conventional breeding programs. Traits such as adaptation to organic management of soil fertility appear to require selection under organic soil conditions for optimal results [23]. Murphy et al. [24] demonstrated that bread wheat genotypes with the highest yields in conventional systems do not also give the highest yields in organic systems. According to the aforementioned researchers, breeding for higher yields in organic systems requires direct selection within organic systems rather than indirect selection in conventional ones.

In general, landraces represent significantly wider genetic variability than modern (elite) cultivars and could contribute to expanding the genetic base of modern cultivars by contributing many important traits to cope with the pressures of changing environments due to climate change [10,17,21,25,26,27,28,29]. Therefore, a thorough investigation of genetic diversity and the preservation of landraces for future generations is extremely important.

From the middle of the last century to today, the yield of wheat has increased 2–2.5 times [1]. Based on Simmonds [30], genotypes contributed 25–40%, culture environment 30–60%, and Genotype × Environment (G × E) interaction the remaining 15–25%. So multi-environment field evaluation is a necessary practice that has been followed for the selection of high-yield best-adapted genotypes. Moreover, considering the effect of G × E in wheat yield per ha increase [30], the study of that parameter is of high importance, since genotype rank changes from one environment to another, or the concept of crossover interaction, hampers the discovery of superior genotypes [31]. Several statistical tools have been proposed for the G × E interpretation and study of crop stability. However, a complete visual evaluation of all aspects of the G × E interaction is possible with the genotype plus genotype by environment (GGE) biplot model [32,33]. This model emphasizes the two components of genotype effects (G) and G × E by creating a biplot of both mean yield performance and stability and removing the noise caused by the environment’s main effect (E). The model measures the distance of a genotype from the ‘ideal genotype’ that holds the concentric center of a set of concentric cycles and combines high productivity and stability. So, the smaller the distance to the “ideal genotype”, the more desirable it is [34].

Currently, wheat cultivation predominantly takes place in conventional high-input systems, particularly in highly developed countries. These systems rely heavily on chemical inputs such as fertilizers, weedicides, and pesticides to achieve high productivity. However, there is a growing trend towards low-input or organic wheat farming in order to reduce cultivation costs and mitigate the adverse environmental impacts associated with conventional systems. Organic farming in wheat agricultural systems involves cultivation without the use of synthetic chemical fertilizers, pesticides, or weedicides. Instead, farm fertility is maintained through practices such as crop rotation with legumes and the incorporation of green manure or animal manure. Organic farming is increasing worldwide. The European Commission (EC) has set a target of converting at least 25% of the EU’s agricultural land under organic farming by 2030 [35]. However, modern commercial varieties are suitable for high-input farming systems, and there is a lack of cultivars adaptable to organic conditions or low-input agriculture. Perhaps, landraces could provide a solution to this problem in wheat farming since they exhibited high adaptability and productivity in low-input farming systems or production under organic conditions.

The primary objective of this study is to investigate the agronomic value and adaptability of landraces in low-input/organic environments compared to commercial cultivars. Additionally, the study aims to identify potential landraces that can serve as starting genetic material in a bread wheat breeding program targeting low-input/organic agricultural systems.

## 2. Results

First Season of Experimentation

**Figure 1:**
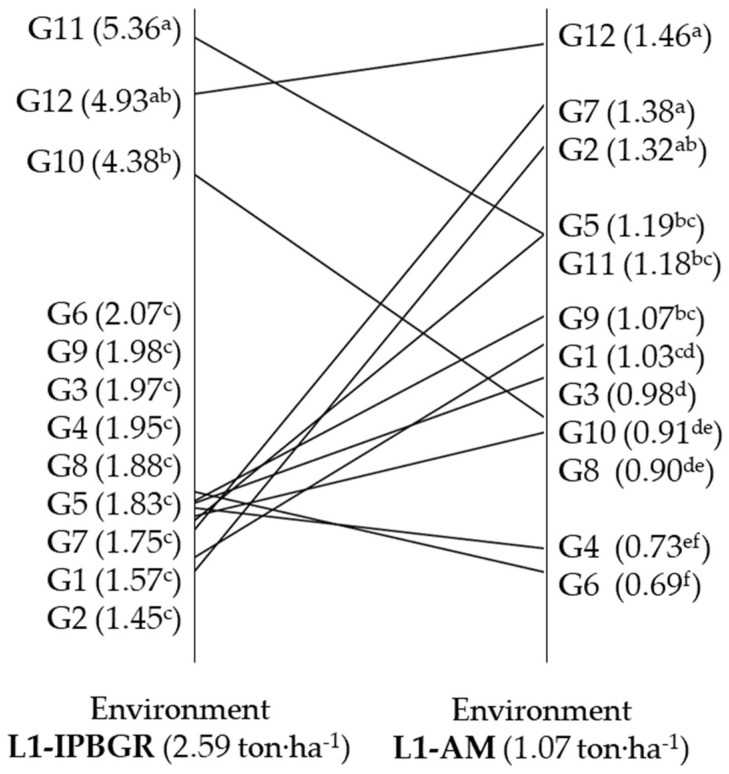
Matching of wheat genotype’s yield in two low-input environments (L1-IPBGR, L1-AM) during the evaluation in 2013–2014 cultivation season. (Means followed by the same letter in each environment are not significantly different based on Tuckey’s test and *p ≤* 0.05).

Analysis of variance (ANOVA) showed statistically significant differences between genotypes and between growing environments for all measured agronomic, morphological, and seed quality traits (Table 1 and Table 2). Additionally, a significant interaction between genotype and environment was found for all traits except the number of seeds per ear (NS). This means that the genotypes behave differently in the different growing environments for all traits except NS. Furthermore, the interaction was quite clear in terms of yield; e.g., the landrace “Hasiko Kritis” (G7) was ranked second in a low-input environment at the Farm of the Agricultural Research Station of Agios Mamas (L1-AM), while it was ranked last in a low-input environment at the Farm of the Institute of Plant Breeding and Genetic Resources (L1-IPBGR) (Figure 1). On the other hand, the commercial cultivar “Gkogkas-2” (G12) showed high stability and ranked first and second in both environments.

More specifically, grain yield (GY) was almost 2.5 times higher, plant height (PH) was ~20 cm longer, ear length either without awns (EL) or with awns (ELA) was 0.6 cm longer, protein content (PC) was ~1.6% higher, gluten index (GI) was 4.4 units higher, sedimentation value (SV) was 2.4 units higher, and falling number (FN) was 77.4 units higher, while the NS was 4.3 units less, and thousand kernel weight (TKW) was 8.0 g less in the L1-IPBGR compared to L1-AM, which is considered a low productivity environment based on previous experimentation of the former Cereal Institute of Greece.

The landraces had almost half the yield (1.55 ton·ha^−1^) compared to commercial cultivars (3.24 ton·ha^−1^). Among the landraces, the landrace “Xilocastro Lamias” (G10) excelled in production with a yield reaching 2.65 ton·ha^−1^, while there were no differences between all other landraces. In addition, the landraces had an increased PH of ~40 cm compared to the commercial cultivars. Only the most productive landrace, G10, had a similar PH to the commercial cultivars. As far as EL is concerned, the highest values (13.9–14.4 cm) had the landraces “4 Kontopouli” (G4), “Mavravani Aetoloakarnanias” (G6), and “18 Kontopouli 16” (G3), while, for NS, the landrace G10 had the highest value, as did the commercial cultivar G12.

PC is considered one of the most important grain quality characteristics. Thus, for PC, the best landraces were G7 and “Atheras Kerkiras” (G1), followed by “Zoulitsa Arkadias” (G2), with about 1.0% higher PC from the best commercial cultivar G12. The highest GI was observed in the commercial cultivar “Yecora E” (G11), while all landraces did not differ from the other commercial cultivar, G12. For SV, the landrace G3 showed the highest value, while for FN, the landrace “Mavragani Argolidas” (G9) had the highest value, with the majority of landraces showing similar values. Finally, the landraces with the highest TKW were “Tsipoura Samou” (G5), G2, G4, G7, and G9.

A high negative correlation (r_pearson_ = −0.946 **) was found between GY and PH (Table 3). This correlation was expected, as tall landraces faced a lodging problem that resulted in a significant reduction in grain yield. The “Green Revolution” in the middle of the last century was based on reducing the pH of wheat varieties and increasing productivity. Furthermore, a high negative correlation was found between PH and GI (r_pearson_ = −0.835 **), NS and TKW (r_pearson_ = −0.798 **), NS and PC (r_pearson_ = −0.710 **), and GI and FN (r_pearson_ = −0.812 **). A medium negative correlation was found between GY and PC (r_pearson_ = −0.658 *) as expected, between GY and EL (r_pearson_ = −0.634 *), and between NS and PH ((r_pearson_ = −0.611 **). On the other hand, a high positive correlation was found between GY and NS (r_pearson_ = 0.721 **), GY and GI (r_pearson_ = 0.745 **), and a moderate positive correlation between PH and EL (r_pearson_ = 0.577 *), PH and PC (r_pearson_ = 0.700 *), and PH and FN (r_pearson_ = 0.592 *).

Second Season of Experimentation

Over-location analysis of variance (ANOVA) showed differences between genotypes and between cultivation environments for all measured agronomic, morphological, and seed quality characteristics (Table 4, Table 5, Table 6 and Tables S1–S5). Additionally, a significant interaction was found for GY, days in heading (HD), EL, and TKW, which means that genotypes showed different behavior in the different evaluation environments. However, no interaction was found for PH and NS, which means that the evaluated genotypes showed similar behavior for those characteristics in the different evaluation environments.

More specifically, the conventional environment at the farm of the Institute of Plant Breeding and Genetic Resources-IPGRB (C2-IPGRB) showed 31.3% higher productivity compared to the organic environment at the same farm (O2-IPGRB) and 11.8% and 97.1%, respectively, compared to the low-input environments at the farms of the IPGRB (L2-IPBGR) and Aristotle University of Thessaloniki-AUTH (L2-AUTH). The field in the AUTH farm is considered to be of very low fertility. The tallest plants (PH) were in the O2-IPGRB environment (96.2 cm), while the shortest (83.7 cm) were in the low-productivity L2-AUTH environment. Moreover, this environment (L2-AUTH) showed a delay of ~3 HD compared to the other environments. The EL in the L2-AUTH and O2-IPGRB environments was 1.0 and 0.8 cm longer, respectively, than the mean value of low-input environments. Ears of wheat plants in the O2-IPGRB environment had 5–8 more seeds (NS) than ears in the other environments. TKW in the low-input environments was ~7–8 g lower in comparison with the mean of organic and conventional environments. The coefficient of variation (CV) for quantitative traits like GY was quite high compared to qualitative traits like TKW and HD. HD is a qualitative characteristic that is controlled by a significantly smaller number of genes compared to GY, so it has a high heritability, which is why it has a very low CV value.

The most productive genotype in the over-environment analysis that included two low-input environments, one conventional and one organic, which was the commercial cultivar “Yecora E” (X5) (2.57 ton·ha^−1^) and the landrace “Xilokastro Lamias” (X4) (2.52 ton·ha^−1^) followed by the commercial cultivar “Panifor” (X7) (2.47 ton·ha^−1^). A significant interaction was found for GY in the over-environment analysis, meaning that an ANOVA of GY in each of the four environments was necessary (Table 6). ANOVA reveals differences between genotypes in each environment. More specifically, in the low-productivity and low-input environment (L2-AUTH), the landrace X4 showed the highest productivity (1.83 ton·ha^−1^) followed by the commercial cultivar X7 (1.66 ton·ha^−1^). The rest of the genotypes had ~50 to 100% less grain yield compared to X4. In the good productivity low-input environment (L2-IPGRB), the range of GY was from 1.19 ton·ha^−1^ [“Mavragani Aetoloakarnanias” (X3)] to 3.16 ton·ha^−1^ (X5). Additionally, high GY was presented by the landraces “Atheras Kerkiras 185” (X1), “Zoulitsa Arkadias” (X2), and X4 and the commercial cultivar X7, which did not differ significantly from X5. In the conventional environment (C2-IPGRB), commercial cultivars X7, “Africa” (X8), and X5 showed the highest values, 3.41, 3.25, and 3.09 ton·ha^−1^, respectively. Of the landraces, only the X4 showed a high GY (3.10 ton·ha^−1^), while the other landraces had ~33–85% lower yield. In the organic environment (O2-IPGRB), the highest productivity was found in the commercial cultivar X5 and the landrace X4. Commercial cultivars X8 and X7 showed ~68% reduction in GY in the organic environment compared to the conventional, while this reduction was half for the landraces. Moreover, one landrace (X1) showed increased GY in the organic environment.

**Figure 2:**
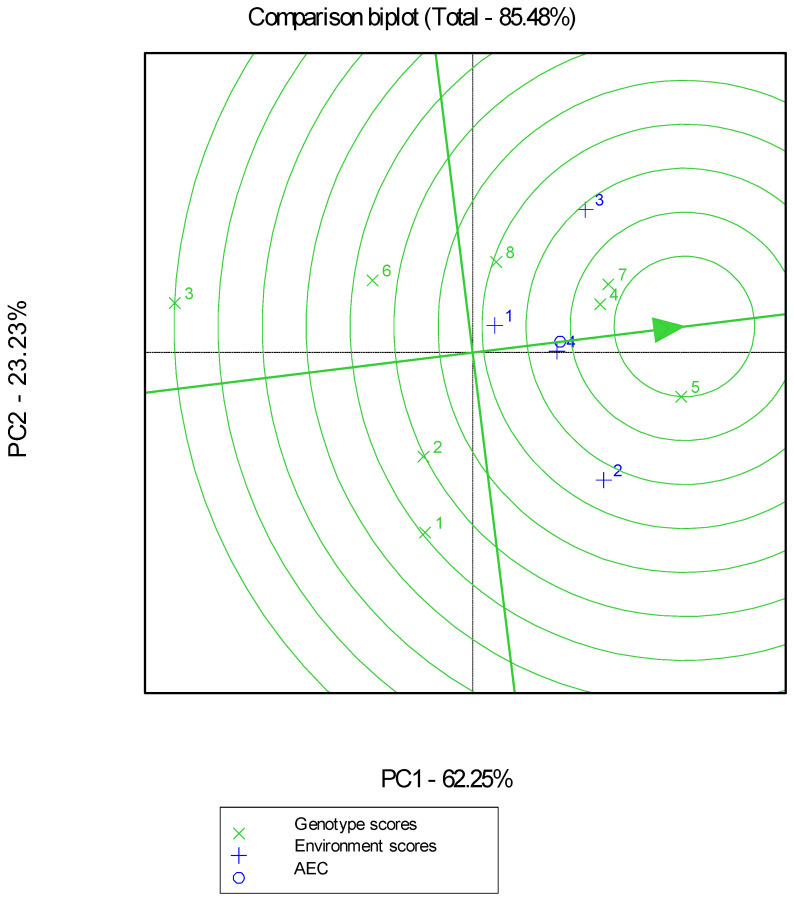
Genotype and genotype by environment (GGE) biplot for GY production of the 8 genotypes evaluated in 4 environments (“×” code corresponds to genotypes and “+” code to environments). Abbreviations: Environments: L2-AUTH (+1), L2-IPGRB (+2), C2-IPGRB (+3), O2-IPGRB (+4); Landraces: “Atheras Kerkiras 185” (X1), “Zoulitsa Arkadias” (X2), “Mavragani Aetoloakarnanias” (X3), “Xilokastro Lamias” (X4); Cultivars: “Yecora E” (X5), “Accor” (X6), “Panifor” (X7), “Africa” (X8).

**Figure 3:**
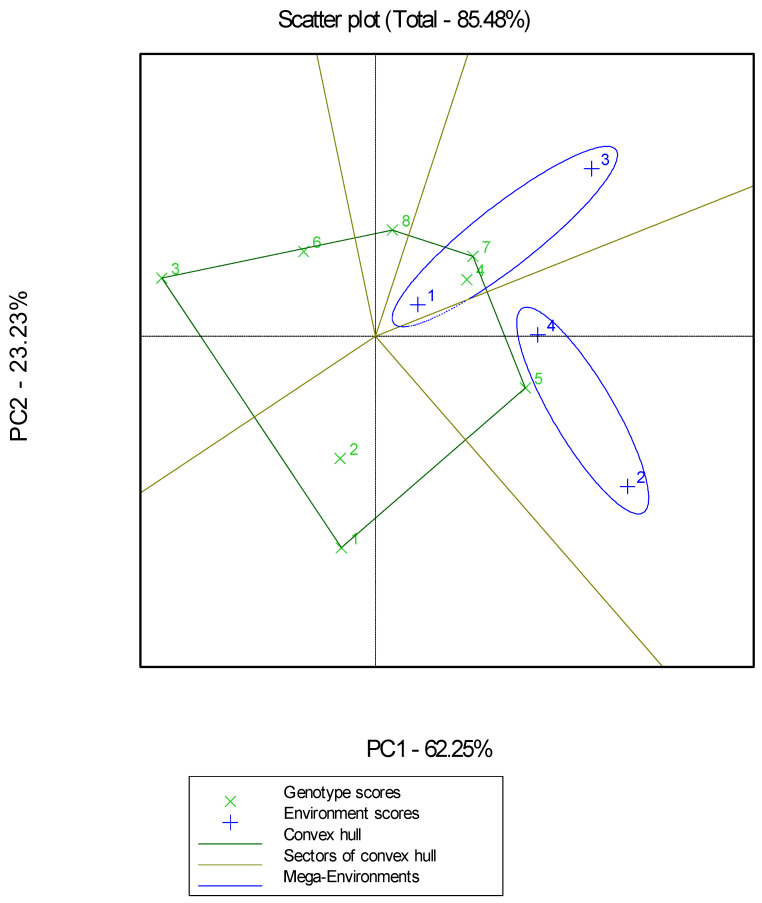
Genotype and genotype by environment (GGE) biplot for GY production of the 8 genotypes evaluated in 4 environments (“×” code corresponds to genotypes and “+” code to environments).

GGE biplot analysis explained 85.48% of the total variability and revealed that the highest GY X5 was placed closest to the point of the “ideal genotype” in terms of performance and stability in all environments (Figure 2). Genotype X5 ranked first in the high-productivity low-input environment (L2-IPGRB) and organic environment (O2-IPGRB) and third in the conventional environment (C2-IPGRB), while it was inferior in the low-productivity and low-input environment (L2-AUTH) ranking fifth. Second-ranked based on GGE biplot analysis for performance and stability were genotypes X4 and X7. More specifically, genotype X4 ranked first in low productivity and low-input environment (L2-AUTH), second in organic environment (O2-IPGRB), and fifth in high productivity and low-input environment (L2-IPGRB). Genotype X7 ranked second in the low-input environment (L2-AUTH) and third in both the low-input, high-productivity environment (L2-IPGRB) and the organic environment (O2-IPGRB). The “which-won-where” projection of the GGE biplot (Figure 3) showed two mega-environments, i.e., L2-AUTH environment (noted as +1) and C2-IPGRB (noted as +3) formed the first mega-environment with X7 and X4 as winning genotypes; high productivity low-input environment (L2-IPGRB) (noted as +2) and organic environment (O2-IPGRB) (noted as +4) formed the second mega-environment with X5 as the winning genotype.

All statistically significant correlations recorded in the second growing season had the same signs as those in the first season, differing only in the strength of the correlations. Thus, PH correlated negatively with GY (r_pearson_ = −0.181 *) and NS (r_pearson_ = −0.426 **), while positively low with HD (r_pearson_ = 0.372 **) and TKW (r_pearson_ = 0.313 **) and positively moderately with the EL (r_pearson_ = 0.710 **) (Table 7). The main difference between the evaluated genotypes of the two growing periods was that in the first period mainly tall landraces were evaluated, while in the second period, four of the eight evaluated genotypes were short commercial bread wheat cultivars. The reduction in the PH of the evaluated genotypes clearly explains the reduction in the strength of the correlations between PH and GY, EL, and NS compared to the first growing season. Moreover, GY did not correlate with the other components of production, which are NS and TKW. The reduced rainfall during April and May 2015 compared to the first period did not allow a satisfactory utilization of the fertilization and grain filling, resulting in the reduction of the value of the negative correlation between GY and EL (r_pearson_ = −0.369 **) and the absence of a significant correlation between GY and NS. Finally, a negative correlation was recorded between HD and GY (r_pearson_ = −0.400 **), HD and TKW (r_pearson_ = −0.397 **), NS and EL (r_pearson_ = −0.455 **) and NS and TKW (r_pearson_ = −0.330 **), while a positive correlation was recorded between EL and HD (r_pearson_ = 0.269 **) and EL and TKW (r_pearson_ = 0.225 *) (Table 7).

## 3. Discussion

Wheat is a crucial crop grown worldwide on 218.5 million hectares and is one of the most important crops in Europe [1]. To promote environmentally friendly agriculture practices, the European Union’s agricultural policy (European Green Deal) prioritizes low-input production methods. A crucial goal of this approach is to increase the percentage of organic farming to 25% of the total agricultural area in the EU by 2030 [35]. It is important to note that economic factors, including the escalating costs of inputs, and geopolitical events such as the Russia-Ukraine war, are prompting low-input/organic agriculture systems [36]. However, there is a lack of information on which genotypes are suitable for cultivation in low-input/organic farming since modern commercial cultivars were developed in high-input farming systems. Cultivation of adaptable varieties is an effective and low-cost agronomic practice for sustainable wheat production under current and future climate change scenarios [28]. The absence of modern wheat cultivars specifically adapted to low-input or organic farming systems has highlighted the significance of conserving and exploiting landraces, which typically possess the adaptability to such agricultural practices [22]. Perhaps, landraces could provide a solution to this problem, which is also characterized by high tolerance to biotic and abiotic stresses, high yield stability, and average yield in low-input environments [4,12,13,14]. The evaluation of agronomic and quality traits in the over-environmental evaluation can be considered a holistic methodology to estimate the productive potential of genotypes under prevailing climatic conditions, thus revealing their suitability for agricultural production. In terms of weather conditions, higher rainfall prevailed in April 2014 in the Thermi environment (L1-IPBGR) compared to the Agios Mamas environment (L1-AM), resulting in a 2.5 higher GY. Genotypes utilized the more fertile environment of L1-IPBGR and produced more tillers per unit area. So in the L1-AM, fewer tillers per unit area means that the ears have more resources to exploit, resulting in more (number of seeds) and larger (thousand kernel weight) seeds per ear.

This is because the grain filling period is defined as critical and has a major impact on wheat yield. Several research studies have found that weather conditions, particularly the amount and distribution of precipitation, have an impact on wheat yield [37,38,39]. Analysis of variance (ANOVA) showed statistically significant differences between genotypes and evaluation environments for GY and all the measured agronomic, morphological, and seed quality traits in both experimental years. Previous bread wheat yield research has also demonstrated the cultivar variation presented in our study [37,39,40,41]. Most landraces, as expected, had higher PH than commercial cultivars, ranging from ~40 cm to 60 cm. However, the landrace with the lowest PH (~90 cm) was among the most productive genotypes, which is connected with a higher harvest index. By studying the grain quality characteristics of the different genotypes in the present study, genetic variability and promising genotypes were identified that can be considered as valuable parent materials to be exploited in bread wheat breeding programs aimed at improving the grain quality characteristics and widening the range for wheat end-users [42,43].

Moreover, wheat landraces are considered an excellent source of variation in a breeding program for tolerance to biotic and abiotic stresses, seed quality characteristics, high yield stability in low-input/organic environments, and numerous other important traits that could help crops withstand the challenges arising from environmental change caused by climate change [4,12,13,14,17,18,25,29,42,43,44,45]. A promising landrace as a starting material for a bread wheat breeding program for yield and yield stability is “Xilokastro Lamias”, which in the first growing season outperformed the rest of the landraces yielding 2.65 ton·ha^−1^, while during the second growing season with a grain yield of 2.57 ton·ha^−1^ gave a similar yield to the two best commercial cultivars. Additionally, as a source of variation, it could contribute to expanding the genetic base of cultivated bread wheat and reduce the erosion of biodiversity, as the new elite wheat cultivars were derived from a narrow germplasm pool [9]. Furthermore, landraces in an autogamous crop like wheat are a mixture of pure lines and could result in the release of genetically stable genotypes or commercial cultivars that may be suitable for organic/low-input environments in a very short time. There are many successful examples of intensive breeding programs where effective plant selection under ultra-low plant density has resulted in the exploitation of landraces/cultivars variation in wheat [46,47] and other autogamous species [48,49] and finally led to genetically stable genotypes in a short time by reducing the breeding program time to half or a third.

The lack of cultivars capable of adapting to organic conditions or low-input agriculture, despite the growing demand for organic farming in the European Union (EU), which has set a target of achieving at least 25% of the EU’s agricultural land under organic farming by 2030 [35], stems from the observation that bread wheat genotypes that excel in conventional systems do not consistently demonstrate the same performance in organic systems. Because of this situation, landraces are emerging as a promising solution in the field of wheat farming. Their proven capacity to display exceptional adaptability and productivity in low-input farming systems or organic conditions positions them as viable alternatives to address this challenge. Considering that, in the first year of experimentation, ten landraces were evaluated in two low-input environments compared to two commercial cultivars. The four best landraces were selected based on yield and seed quality characteristics and evaluated during the second year of experimentation in four environments, including two low-input, one organic, and one conventional. Conducting multi-environmental field evaluations is an essential approach that has been implemented to select wheat genotypes that combine high and stable yields. Considering the significant impact of genotype-by-environment interactions (G × E) on increasing wheat yield per hectare [30], a thorough investigation of this parameter is of paramount importance. The dynamic nature of G × E interactions causes variations in the ranking of genotypes across different environments, thereby presenting a formidable challenge in identifying superior genotypes [31]. In our research, the study of G × E interactions plays a crucial role in comprehending how genotypes perform under low-input/organic/conventional environmental conditions and are vital for the efficient identification and selection of superior genotypes. GGE biplot analysis revealed that environments L2-AUTH and C2-IPGRB formed the first mega-environment with genotypes “Panifor” (X7) and “Xilokastro Lamias” (X4) as winners, and L2-IPGRB and O2-IPGRB formed the second mega-environment with “Yecora E” (X5) as the winner genotype. GGE biplot analysis revealed landraces (X4) and commercial cultivars (X5, X7) that could be used directly in low-input/organic wheat agricultural systems. It is important to note that two of the most productive and stable genotypes had the shortest heading date in their group; e.g., X4 had the shortest HD between landraces and similarly, X7 in the cultivar group. The importance of the connection of the shortest HD with the higher GY, is also shown in the negative correlation of HD with the GY. Other researchers have successfully used GGE biplot analysis in cereals to select high-yielding and stable genotypes in bread wheat [50,51] and other crops [52,53,54]. Conventional management yielded ~47% more grain compared to the mean yield of the low-input/organic environment. Similar rates (~30% to 60%) have been found by other researchers [55,56] comparing conventional versus organic evaluation environments. One low-input environment (L2-IPGRB) was superior to organic (~17%), while the other was inferior (L2-AUTH) (~33%). Other studies showed that grain yield under organic management increased compared to that under low inputs [57], while in others, it decreased [58]. Moreover, the reduction of GY between conventional and organic environments was 45% for commercial cultivars, while it was half for landraces, confirming their adaptability to organic agriculture.

## 4. Materials and Methods

### 4.1. Genetic Material and Environments

First Year of Experimentation (Two Environments)

**Figure 4:**
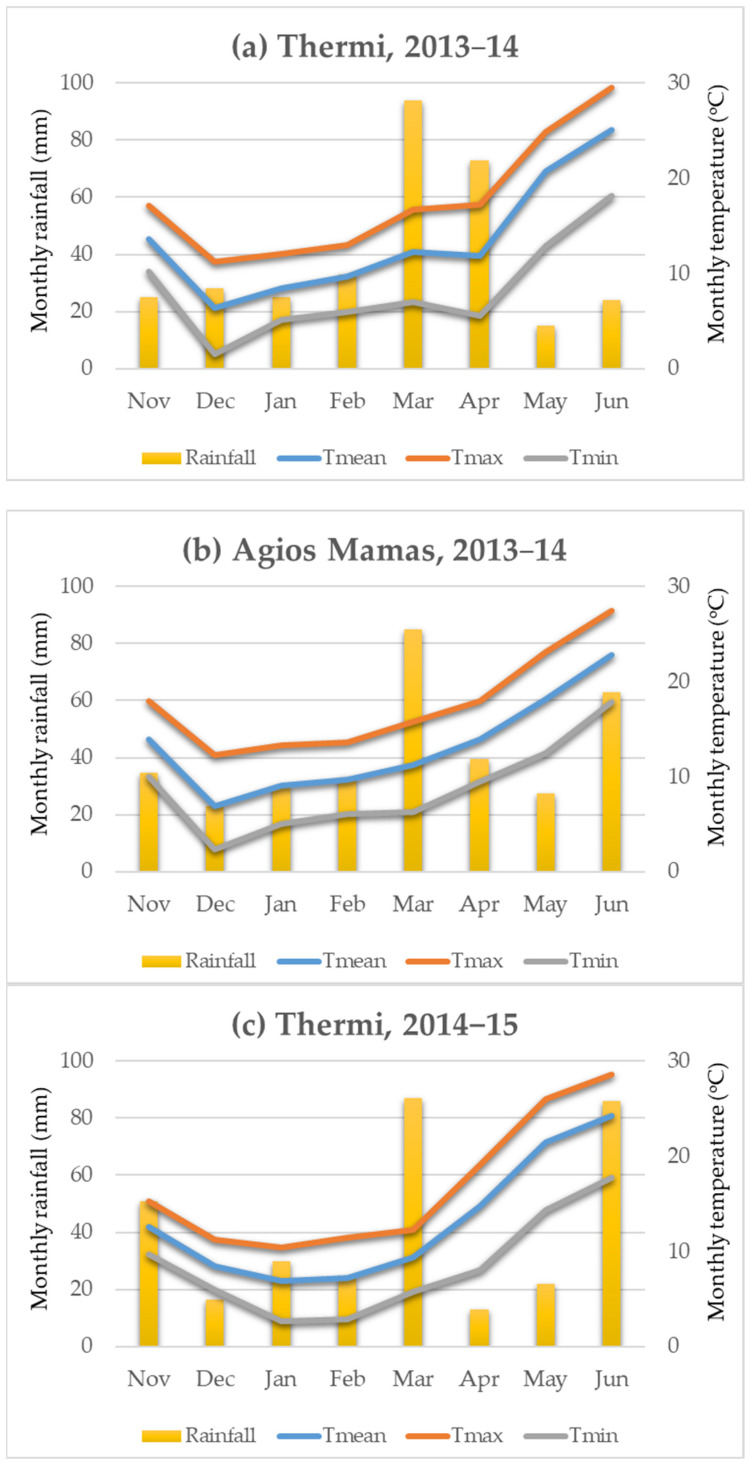
Basic weather data (mean, maximum, and minimum monthly temperature in °C and rainfall in mm) based on daily records, for years: (**a)** 2013–2014 at Thermi (IPBGR farm); (**b**) 2013–2014 at the Agios Mamas farm; (**c**) 2014–2015 at Thermi (IPBGR farm and AUTH farm).

Nine bread wheat landraces, namely “Atheras Kerkiras 185” (G1), “Zoulitsa Arkadias” = (G2), “18 Kontopouli 16” (G3), “4 Kontopouli” (G4), “Tsipoura Samou” (G5), “Mavragani Aetoloakarnanias” (G6), “Hasiko Kritis” (G7), “Asprostaro Larisas” (G8), and “Mavragani Argolidas” (G9), one improved wheat landrace “Xilokastro Lamias” (G10), and two commercial cultivars “Yecora E” (G11), and “Gkogkas 2” (G12) were evaluated in two low-inputs field experiments, during the first growing season of 2013–2014. The first evaluation environment was at the Farm of the Institute of Plant Breeding and Genetic Resources (L1-IPBGR) in Thermi (40°54′ N, 23°00′ E), and the second evaluation environment was at the Farm of the Agricultural Research Station of Agios Mamas (L1-AM) (40°24′ N 23°33′ E) (Table 8 and Table 9, Figure 4). The same agricultural practices were applied in both fields in order to evaluate landrace adaptability in low-input environments. Local landraces G1–G9 were cultivated in different regions throughout Greece before 1923, as recorded by Papadakis [59] and landrace G10 is a genotype selected and improved under low-input conditions.

Second Year of Experimentation (Four Environments)

For the next growing season, from the 10 bread wheat landraces of the first year, 4 landraces, “Atheras Kerkiras 185” (X1), “Zoulitsa Arkadias” (X2), “Mavragani Aetoloakarnanias” (X3), and “Xilokastro Lamias” (X4), were selected according to the agronomic and quality traits evaluation of the 2013–2014 growing season. Among the 10 landraces, the landrace X4 excelled in GY, NS, HW, GI, and FN, while having the lowest value in PH. Landrace L3 had better EL, PC, GI, SV, and FN. Landrace X2 had good values in TKW, PC, and GI. The landrace X1 had good values for ELA, HW, PC, GI, and FN. Additionally, experimentation included four important commercial bread wheat cultivars “Yecora E” (X5), “Accor” (X6), “Panifor” (X7), and “Africa” (X8).

In the second growing season, in order to evaluate the behavior of the selected landraces in different cropping systems (low inputs, conventional, organic), the experimental fields were established in neighboring fields with similar soil fertility status (Kirchmann et al. 2016) and the inputs were varied. The experimentation included evaluation in four field environments (Table 8 and Table 9, Figure 4):

- L2-AUTH: Low-input environment at the Farm of Aristotle University of Thessaloniki (AUTH);
- L2-IPGRB: Low-input environment at the Farm of IPBGR;
- C2-IPGRB: Conventional field at the Farm of IPBGR;
- O2-IPGRB: Organic field at the Farm of IPBGR, in which a rotation program with vetch and incorporation of green manure every two years is applied.
- It was established randomized complete block design, with four replications on plots of 3.5 m^2^ in all experimental fields in both cropping seasons (2013–2014, 2014–2015). Moreover, all experiments were established in the middle of November, while the harvest was at the end of June.

### 4.2. Agronomic and Morphological Ear Characteristics

In total, 20 plants were selected randomly from the non-border lines of each plot, and plant height (PH) (cm), ear length (EL) without and with awns (ELA) (cm), and number of seeds (NSD) per ear were measured. Additionally, the grain yield (GY) of each plot was estimated and expressed as ton·ha^−1^.

### 4.3. Seed Quality Characteristics

Thousand kernel weight (TKW) (g) was measured in four samples for each plot. The test weight, or hectoliter weight (HW) (Kg.hL^−1^), was measured with KERN ALBSTADT Germany in two samples for each plot. Protein content (PC) was analyzed with Perten’s Inframatic 8620 infrared analyzer using the Kjeldahl method (N × 5.7) (ICC METHOD 159). The gluten index (GI) was determined using a Perten Glutomatic instrument (ICC METHOD 155 & 158). The Falling Number (FN) indirectly calculates the amylase activity resulting from the presence of germinated grains (ICC METHOD 107/1). The Sedimentation Value (SV) according to Zeleny is affected by both the quality and quantity of gluten and is taken as a measure of baking quality (ICC METHOD 116/1).

### 4.4. Statistical Analysis

SPSS software package (ver. 18. SPSS Inc., Chicago, IL, USA) was used for the over-location one-factor (Genotypes) analysis of variance (ANOVA) of the experiments conducted: (1) during the 2013–2014 cultivation period and (2) during the 2014–2015 cultivation period. The significance level of all hypotheses tested was pre-set at *p* ≤ 0.05, using Tuckey’s test (*p* < 0.05). In addition, Pearson correlation coefficients were estimated.

To determine mean performance in combination with stability across environments, a genotype and genotype × environment (GGE) biplot analysis [32,33] was done with normalized data using Genstat (13) [60]. This model measures the distance of each genotype from the ‘ideal genotype’, i.e., the virtual genotype that has the best combination of mean performance and stability.

## 5. Conclusions

Significant variability for agronomic and seed quality characteristics was found in landraces through field evaluation in low-input/organic/conventional environments, showing that it could be an excellent source of variation in a breeding program targeting high and stable production and high-quality product in a low-input/organic environment. Moreover, landraces could help expand the genetic base of cultivated bread wheat and reduce biodiversity erosion, helping crops withstand the challenges of low-input/organic agriculture. Landrace “Xilokastro Lamias” (X4) was a promising starting material in a bread wheat breeding program for its high and stable yield. GGE biplot analysis revealed a landrace (“Xilokastro Lamias”) and commercial cultivars (“Yecora E” and “Panifor”) that could be directly used in low-input/organic wheat agricultural systems for high and stable productivity. Finally, the reduction in grain yield between conventional and organic environments was observed to be 45% for commercial cultivars, while it was only half for landraces. This finding confirms the adaptability of landraces to organic agriculture.

## Figures and Tables

**Table 1:** Over location analysis of variance (ANOVA) of bread wheat landraces and cultivars and their interaction with location, for 10 agronomic, morphological, and seed quality characteristics, during first cultivation period (2013–2014).

Source of Variation	df	GY	PH	EL	ELA	NS	TKW	PC	GI	SV	FN
E	1	**	**	**	**	**	**	**	**	**	**
G	11	**	**	**	**	**	**	**	**	**	**
G × E	11	**	**	*	*	ns	**	**	*	*	**
Blocks	3	ns	*	ns	ns	ns	ns	ns	ns	ns	ns
error	69										
CV%		12.7	4.5	5.3	5.5	13.0	5.6	3.2	14.9	12.01	6.1

F-probability values: * *p* ≤ 0.05; ** *p* ≤ 0.01; ns = not significant. Abbreviations: Environments (E), Genotypes (G), Coefficient of Variation (CV), Grain yield (GY), Plant height (PH), Ear length without awns (EL), Ear length with awns (ELA), Number of seeds per ear (NS), Thousand kernel weight (TKW), Protein content (PC), Gluten index (GI), Sedimentation value (SV), Falling number (FN).

**Table 2:** Means of 10 agronomic, morphological, and seed quality characteristics per environment and bread wheat genotype.

2013–2014	GY	PH	EL	ELA	NS	TKW	PC	GI	SV	FN
Environments										
L1-IPBGR	2.59 a†	126.5 a	12.3 a	16.8 a	37.9 b	36.4 b	16.01 a	49.2 a	35.8 a	461.2 a
L-AM	1.07 b	107.3 b	11.7 b	16.2 b	42.2 a	44.4 a	14.39 b	44.8 b	33.4 b	383.8 b
Genotypes										
G1	1.30 c	129.8 ab	11.6 de	18.0 abc	35.5 bcd	38.4 cd	15.90 a	44.6 bc	30.6 def	445.6 a
G2	1.39 c	122.7 bc	12.9 bc	18.9 b	31.1 d	44.8 a	15.79 ab	47.6 bc	35.3 bcde	389.4 c
G3	1.48 c	129.2 ab	13.9 ab	19.3 a	41.1 bc	36.1 de	15.06 bcd	43.1 bc	44.5 a	450.6 a
G4	1.34 c	123.8 bc	14.4 a	14.5 f	32.5 cd	44.5 a	15.19 abc	38.1 c	32.7 cde	400.5 bc
G5	1.51 c	119.4 c	10.9 ef	16.3 de	39.3 bcd	42.9 ab	14.82 cd	41.9 bc	28.6 ef	446.4 a
G6	1.38 c	134.1 a	14.1 a	14.1 f	35.4 bcd	41.6 bc	15.13 abcd	44.8 bc	40.1 ab	431.4 abc
G7	1.57 c	129.8 ab	10.3 f	16.8 cd	34.2 cd	41.7 abc	15.93 a	38.7 c	25.6 f	437.1 ab
G8	1.39 c	128.5 ab	12.6 cd	18.0 abc	43.6 b	39.3 bcd	15.32 abc	46.9 bc	36.0 bcd	439.0 ab
G9	1.53 c	123.4 bc	12.0 cde	17.3 bcd	35.3 bcd	42.8 ab	15.43 abc	41.7 bc	35.8 bcd	465.6 a
G10	2.65 b	92.1 d	10.4 f	15.1 ef	56.1 a	34.3 e	14.31 d	53.1 b	34.0 bcde	450.4 a
G11	3.27 a	74.3 e	10.9 ef	15.2 ef	40.7 bc	41.7 abc	14.65 cd	75.7 a	34.1 bcde	264.9 d
G12	3.20 a	96.1 d	9.9 f	14.6 f	55.9 a	37.0 de	14.86 cd	47.9 bc	37.9 abc	449.1 a

† Means followed by the same letter in a column are not significantly different (Tuckey’s test, *p* ≤ 0.05). Abbreviations: Environments: 1. Low-input environment at Farm of the Agricultural Research Station of Agios Mamas (L1-AM), 2. Low-input environment at Farm of the Institute of Plant Breeding and Genetic Resources (L1-IPBGR); Genotypes: “Atheras Kerkiras 185” (G1), “Zoulitsa Arkadias” (G2), “18 Kontopouli 16” (G3), “4 Kontopouli” (G4), “Tsipoura Samou” (G5), “Mavragani Aetoloakarnanias” (G6), “Hasiko Kritis” (G7), “Asprostaro Larisas” (G8), “Mavragani Argolidas” (G9), “Xilokastro Lamias” (G10), “Yecora E”(G11), “Gkogkas 2” (G12).

**Table 3:** Correlation between genotype values of ten agronomic, morphological, and seed quality characteristics.

	GY	PH	EL	ELA	NS	TKW	PC	GI	SV	FN
GY	1	−0.946 **	−0.634 *	−0.499	0.721 **	−0.398	−0.658 *	0.745 **	0.082	−0.452
PH		1	0.577 *	0.456	−0.611 *	0.267	0.700 *	−0.835 **	0.015	0.592*
EL			1	0.175	−0.553	0.321	0.211	−0.317	0.512	0.003
ELA				1	−0.322	−0.031	0.551	−0.226	0.109	0.220
NS					1	−0.798 **	−0.710 **	0.316	0.238	0.175
TKW						1	0.423	−0.147	−0.360	−0.343
PC							1	−0.497	−0.298	0.196
GI								1	0.109	−0.812 **
SV									1	0.067
FN										1

* Significant correlation at 0.05 level of probability. ** Significant correlation at 0.01 level of probability.

**Table 4:** Over location ANOVA of bread wheat landraces and cultivars and their interaction with location, for seven agronomic, morphological, and seed quality characteristics, during the second cultivation season (2014–2015).

Source of Variation	df	GY	PH	HD	EL	NS	TKW
Environments (E)	3	**	**	**	**	**	**
Genotypes (G)	7	**	**	**	**	**	**
G × E	21	**	ns	**	**	ns	*
Blocks	3	*	*	ns	ns	ns	*
error	93						
CV%		17.1	9.8	0.6	6.3	18.3	10.6

F-probability values: * *p* ≤ 0.05; ** *p* ≤ 0.01; ns = not significant. Abbreviation: Days in heading (HD).

**Table 5:** Means of six agronomic, morphological, and seed quality characteristics per environment and bread wheat landrace and cultivar.

Second Season	GY	PH	HD	EL	NS	TKW
Environments						
L2-AUTH	1.37 d†	86.5 bc	135.5 a	10.45 a	40.9 ab	33.5 c
L2-IPGRB	2.41 b	83.7 d	132.0 b	9.31 b	37.0 b	37.3 b
C2-IPGRB	2.70 a	91.8 ab	132.2 b	9.66 b	38.2 b	40.6 a
O2-IPGRB	2.05 c	96.2 a	132.5 b	10.29 a	45.2 a	42.3 a
Genotypes						
-Landraces						
X1	2.01 c	120.9 a	136.8 a	10.9 b	33.7 de	36.4 cd
X2	1.93 c	115.7 a	136.6 a	11.3 ab	29.3 e	42.2 ab
X3	1.48 d	114.9 a	135.3 b	11.8 a	28.2 e	41.8 ab
X4	2.52 a	87.4 b	131.5 d	9.2 d	55.0 ab	38.0 bc
-Commercial Cultivars						
X5	2.57 a	67.5 c	134.0 c	8.6 de	47.4 bc	32.7 d
X6	1.97 c	63.5 c	136.9 a	8.4 e	55.6 a	32.4 d
X7	2.47 ab	64.7 c	126.3 e	10.0 c	33.0 de	41.0 ab
X8	2.10 bc	81.8 b	126.9 e	9.2 d	40.5 cd	42.9 a

† Means followed by the same letter in a column are not significantly different (Tuckey’s test, *p* ≤ 0.05). Abbreviations: Environments: 1. Low-input environment, at the Farm of Aristotle University of Thessaloniki (L2-AUTH), 2. Low-input environment, at the Farm of IPBGR (L2-IPGRB), Conventional environment at the Farm of IPBGR (C2-IPGRB), Organic environment at the Farm of IPBGR (O2-IPGRB); Landraces: “Atheras Kerkiras 185” (X1), “Zoulitsa Arkadias” (X2), “Mavragani Aetoloakarnanias” (X3), “Xilokastro Lamias” (X4); Cultivars: “Yecora E” (X5), “Accor” (X6), “Panifor” (X7), “Africa” (X8).

**Table 6:** ANOVA and means of bread wheat landraces and cultivars in each of the four evaluation environments for GY, during the second cultivation season (2014–2015).

Second Season		L2-AUTH	L2-IPGRB	C2-IPGRB	O2-IPGRB
	df				
Genotypes	7	**	**	**	**
Blocks	3	**	**	*	**
error	21				
CV%		12.2	14.7	9.5	14.5
Genotypes					
-Landraces					
X1		1.37 bcd†	2.82 ab	1.84 d	2.02 bc
X2		1.10 d	2.72 ab	2.32 cd	1.59 c
X3		1.22 cd	1.19 d	1.97 d	1.55 c
X4		1.83 a	2.60 abc	3.10 ab	2.56 ab
-Commercial Cultivars					
X5		1.25 cd	3.16 a	3.09 ab	2.79 a
X6		1.55 abc	1.79 cd	2.59 bc	1.97 bc
X7		1.66 ab	2.76 ab	3.41 a	2.04 bc
X8		0.99 d	2.26 bc	3.25 a	1.92 bc

F-probability values: * *p* ≤ 0.05; ** *p* ≤ 0.01. † Means followed by the same letter in a column are not significantly different (Tuckey’s test, *p* ≤ 0.05).

**Table 7:** Correlation between genotype means of 6 agronomic, morphological, and seed quality characteristics.

	GY	HD	PH	EL	NS	TKW
GY	1	−0.400 **	−0.181 *	−0.369 **	0.154	0.122
HD		1	0.372 **	0.269 **	0.015	−0.397 **
PH			1	0.710 **	−0.426 **	0.313 **
EL				1	−0.455 **	0.225 *
NS					1	−0.330 **
TKW						1

* Significant correlation at 0.05 level of probability. ** Significant correlation at 0.01 level of probability.

**Table 8:** Soil characteristics of the farms where the experiments were conducted in 2013–2014 and 2014–2015 cropping seasons.

Soil Characteristics	L1-IPBGR	L1-AM	L2-AUTH	L2-IPBGR	C2-IPBGR	O2-IPBGR
Textural Class	L	C	L	L	L	L
Sand (%)	30	30	48	50	48	46
Clay (%)	24	44	20	18	20	22
Silt (%)	46	26	32	32	32	32
pH	7.74	8.04	7.85	7.89	8.00	8.11
EC (mS/cm)	0.596	0.920	0.834	0.503	0.483	0.508
Organic matter (%)	2.54	2.50	1.56	2.37	1.88	2.99
CaCO_3_ (%)	1.80	5.30	5.00	3.00	3.80	7.80
NO_3_ (mg/L)	73.32	74.32	29.46	74.21	27.30	134.16
Nitrogen nitrate	16.56	16.65	6.65	16.68	6.16	30.29
P (mg/L)	4.57	4.13	31.93	31.84	30.02	67.31
K (mg/L)	93	101	377	715	987	1349
Mg^2+^ exchangeable (mg/L)	259	479	416	200	254	309
Ca^2+^ exchangeable (mg/L)	>2000	>2000	>2000	>2000	>2000	>2000
Fe (mg/L)	4.19	4.36	7.15	3.51	3.17	3.05
Zn (mg/L)	0.47	0.18	1.09	0.85	1.11	1.32
Mn (mg/L)	7.15	5.39	5.08	7.53	8.86	10.23
Cu (mg/L)	2.93	1.06	1.66	1.79	2.24	2.54
B (mg/L)	0.46	0.48	0.14	0.16	0.22	0.45

**Table 9:** Details of field operations regarding planting rate, seed treatment, tillage, starter fertilizer, N fertilizer, weed control, and plant disease control practices for bread wheat genotypes in conventional, low-input, and organic cropping systems in 2013–2014 and 2014–2015 cropping seasons.

Descriptor	Conventional	Low-Input	Organic
Planting rate (seeds/m^2^)	~400	~400	~400
Seed Treatment	None	None	None
Tillage	Yes	Yes	Yes
Starter Fertilizer (source)	(250 Kg·ha^−1^) (20-10-0)	(200 Kg·ha^−1^) (20-10-0)	None
Spring application of N fertilizer (source)	67 Kg·N·ha^−1^(33.5-0-0)	None	None
Weed Control	Iodosulfuron-methyl-sodium + mesosulfuron-methyl hiencarbazone + methyl	Iodosulfuron-methyl-sodium + mesosulfuron-methyl hiencarbazone + methyl	None
Plant Disease Control	None	None	None

## Data Availability

Not applicable.

## Supplementary Materials

The following supporting information can be downloaded at: https://www.mdpi.com/article/10.3390/plants12132561/s1, Table S1: ANOVA and means of bread wheat landraces and cultivars in each of the four evaluation environments for HD, during the 2nd cultivation season (2014–2015); Table S2: ANOVA and means of bread wheat landraces and cultivars in each of the four evaluation environments for PH, during the 2nd cultivation season (2014–2015); Table S3: ANOVA and means of bread wheat landraces and cultivars in each of the four evaluation environments for EL, during the 2nd cultivation season (2014–2015); Table S4: ANOVA and means of bread wheat landraces and cultivars in each of the four evaluation environments for NS, during the 2nd cultivation season (2014–2015); Table S5: ANOVA and means of bread wheat landraces and cultivars in each of the four evaluation environments for TKW, during the 2nd cultivation season (2014–2015).

## References

[1] FAOSTAT. https://www.fao.org/faostat/en/#data/QCL.

[2] Peng J.H., Sun D., Nevo E. (2011). Domestication evolution, genetics and genomics in wheat. Mol. Breed..

[3] Beres B.L., Rahmani E., Clarke J.M., Grassini P., Pozniak C.J., Geddes C.M., Porker K.D., May W.E., Ransom J.K. (2020). A Systematic Review of Durum Wheat: Enhancing Production Systems by Exploring Genotype, Environment, and Management (G × E × M) Synergies. Front. Plant Sci..

[4] Xynias I.N., Mavromatis A.G., Korpetis E.G., Pankou C.I., Kozub N.O. (2019). Description and Characterization of Hellenic Wheat Germplasm for Agronomical and Seed Quality Parameters Using Phenotypical, Biochemical and Molecular Approaches. Cytol. Genet..

[5] Martínez-Moreno F., Solís I., Noguero D., Blanco A., Özberk İ., Nsarellah N., Elias E., Mylonas I., Soriano J.M. (2020). Durum Wheat in the Mediterranean Rim: Historical Evolution and Genetic Resources. Genet. Resour. Crop Evol..

[6] Grundas S.T., Caballero B., Trugo L., Finglas P.M. (2003). Wheat: The crop. Encyclopedia of Food Sciences and Nutrition.

[7] Leff B., Ramankutty N., Foley J.A. (2004). Geographic distribution of major crops across the world. Glob. Biogeochem. Cycles.

[8] Hellenic Statistical Authority. https://www.statistics.gr/en/statistics/-/publication/SPG06/-.

[9] Newton A.C., Akar T., Baresel J.P., Bebeli P.J., Bettencourt E., Bladenopoulos K.V., Czembor J.H., Fasoula D.A., Katsiotis A., Koutis K. (2010). Cereal landraces for sustainable agriculture. A review. Agron. Sustain. Dev..

[10] Dwivedi S.L., Ceccarelli S., Blair M.W., Upadhyaya H.D., Are A.K., Ortiz R. (2016). Landrace Germplasm for Improving Yield and Abiotic Stress Adaptation. Trends Plant Sci..

[11] Villa T.C.C., Maxted N., Scholten M., Ford-Lloyd B. (2005). Defining and identifying crop landraces. Plant Genet. Res..

[12] Zeven A.C. (1998). Landraces: A review of definitions and classifications. Euphytica.

[13] Balfourier F., Roussel V., Strelchenko P., Exbrayat-Vinson F., Sourdille P., Boutet G., Koenig J., Ravel C., Mitrofanova O., Beckert M. (2007). A worldwide bread wheat core collection arrayed in a 384-well plate. Theor. Appl. Genet..

[14] Shlibak A.A., Örgeç M., Zencirci N., Zencirci N., Baloch F.S., Habyarimana E., Chung G. (2021). Wheat Landraces Versus Resistance to Biotic and Abiotic Stresses. Wheat Landraces.

[15] Brown A.H.D. (1978). Isozymes, plant population genetic structure and genetic conservation. Theoret. Appl. Genet..

[16] Roupakias D. (2010). Plant Breeding.

[17] Lopes M.S., El-Basyoni I., Baenziger P.S., Singh S., Royo C., Ozbek K., Aktas H., Ozer E., Ozdemir F., Manickavelu A. (2015). Exploiting genetic diversity from landraces in wheat breeding for adaptation to climate change. J. Exp. Bot..

[18] Adhikari S., Kumari J., Jacob S.R., Prasad P., Gangwar O.P., Lata C., Thakur R., Singh A.K., Bansal R., Kumar S. (2022). Landraces-potential treasure for sustainable wheat improvement. Genet. Resour. Crop Evol..

[19] Wood D., Lenné J.M. (1997). The conservation of agrobiodiversity on-farm: Questioning the emerging paradigm. Biodivers. Conserv..

[20] Xynias I.N., Mylonas I., Korpetis E.G., Ninou E., Tsaballa A., Avdikos I.D., Mavromatis A.G. (2020). Durum Wheat Breeding in the Mediterranean Region: Current Status and Future Prospects. Agronomy.

[21] Wingen L.U., West C., Leverington-Waite M., Collier S., Orford S., Goram R., Yang C.Y., King J., Allen A.M., Burridge A. (2017). Wheat Landrace Genome Diversity. Genetics.

[22] Koutis K. (2011). Evaluation and Utilization of Wheat Landraces in Conditions of Reduced Inputs. Ph.D. Thesis.

[23] Lammerts van Bueren E.T., Struik P.C., Jacobsen E. (2002). Ecological concepts in organic farming and their consequences for an organic crop ideotype. NJAS—Wagen. J. Life Sc..

[24] Murphy K.M., Campbell K.G., Lyon S.R., Jones S.S. (2007). Evidence of varietal adaptation to organic farming systems. Field Crops Res..

[25] Reynolds M., Dreccer F., Trethowan R. (2007). Drought-adaptive traits derived from wheat wild relatives and landraces. J. Exp. Bot..

[26] Dotlačil L., Hermuth J., Stehno Z., Dvořáček V., Bradová J., Leišová L. (2010). How can wheat landraces contribute to present breeding?. Czech J. Genet. Plant Breed..

[27] Abu-Zaitoun S.Y., Chandrasekhar K., Assili S., Shtaya M.J., Jamous R.M., Mallah O.B., Nashef K., Sela H., Distelfeld A., Alhajaj N. (2018). Unlocking the Genetic Diversity within A Middle-East Panel of Durum Wheat Landraces for Adaptation to Semi-arid Climate. Agronomy.

[28] Mylonas I., Stavrakoudis D., Katsantonis D., Korpetis E., Ozturk M., Gul A. (2020). Better farming practices to combat climate change. Climate Change and Food Security with Emphasis on Wheat.

[29] Marone D., Russo M.A., Mores A., Ficco D.B.M., Laidò G., Mastrangelo A.M., Borrelli G.M. (2021). Importance of Landraces in Cereal Breeding for Stress Tolerance. Plants.

[30] Simmonds N.W. (1981). Genotype (G), environment (E) and GE components of crop yields. Exper. Agric..

[31] Baker R.J. (1988). Tests for crossover genotype-environmental interactions. Can. J. Plant Sci..

[32] Yan W. (2001). GGEBiplot-A Windows application for graphical analysis of multi-environment trial data and other types of two-way data. Agron. J..

[33] Yan W. (2002). Singular-value partition in biplot analysis of multienvironment trial data. Agron. J..

[34] Yan W., Kang M.S., Ma B., Woods S., Cornelius P.L. (2007). GGE biplot vs. AMMI analysis of genotype-by-environment data. Crop Sci..

[35] European Commission. https://agriculture.ec.europa.eu/farming/organic-farming/organic-action-plan_en.

[36] Ben Hassen T., El Bilali H. (2022). Impacts of the Russia-Ukraine War on Global Food Security: Towards More Sustainable and Resilient Food Systems?. Foods.

[37] Feledyn-Szewczyk B., Cacak-Pietrzak G., Lenc L., Stalenga J. (2020). Rating of Spring Wheat Varieties (*Triticum aestivum* L.) According to Their Suitability for Organic Agriculture. Agronomy.

[38] Rozbicki J., Ceglińska A., Gozdowski D., Jakubczyk M., Cacak-Pietrzak G., Mądry W., Golba J., Piechociński M., Sobczyński G., Studnicki M. (2015). Influence of the cultivar, environment and management on the grain yield and bread-making quality in winter wheat. J. Cer. Sci..

[39] Zargar M., Polityko P., Pakina E., Bayat M., Vandyshev V., Kavhiza N., Kiselev E. (2018). Productivity, quality and economics of four spring wheat (*Triticum aestivum* L.) cultivars as affected by three cultivation technologies. Agron. Res..

[40] Studnicki M., Wijata M., Sobczyński G., Samborski S., Rozbicki J. (2018). Assessing grain yield and quality traits stability of spring wheat cultivars at different crop management levels. Cereal Res. Commun..

[41] Mäder P., Hahn D., Dubois D., Gunst L., Alföldi T., Bergmann H., Oehme M., Amadò R., Schneider H., Graf U. (2007). Wheat quality in organic and conventional farming: Results of 21-year field experiment. J. Sci. Food Agric..

[42] Guzman C., Ammar K., Govindan V., Singh R. (2019). Genetic improvement of wheat grain quality at CIMMYT. Front. Agric. Sci. Eng..

[43] López-Fernández M., Pascual L., Faci I., Fernández M., Ruiz M., Benavente E., Giraldo P. (2021). Exploring the End-Use Quality Potential of a Collection of Spanish Bread Wheat Landraces. Plants.

[44] Casañas F., Simó J., Casals J., Prohens J. (2017). Toward an evolved concept landrace. Front. Plant Sci..

[45] Bladenopoulos K.V., Ninou E.G., Tsochatzis E.D., Mylonas I.G., Hasunuma K. (2014). Organic Breeding and Cultivation of Barley. Effects on Physical and Chemical Properties. Barley: Physical Properties, Genetic Factors and Environmental Impacts on Growth.

[46] Ninou E.G., Mylonas I.G., Tsivelikas A., Ralli P., Dordas C., Tokatlidis I.S. (2014). Wheat Landraces Are Better Qualified as Potential Gene Pools at Ultra spaced Rather than Densely Grown Conditions. Sci. World J..

[47] Ninou E., Mylonas I., Karagianni I., Michailidou S., Tsivelikas A., Sistanis I., Avdikos I., Korpetis E., Papathanasiou F. (2022). Utilization of Intra-Cultivar Variation for Grain Yield and Protein Content within Durum Wheat Cultivars. Agriculture.

[48] Ninou E., Papathanasiou F., Vlachostergios D.N., Mylonas I., Kargiotidou A., Pankou C., Papadopoulos I., Sinapidou E., Tokatlidis I. (2019). Intense Breeding within Lentil Landraces for High-Yielding Pure Lines Sustained the Seed Quality Characteristics. Agriculture.

[49] Koutsika-Sotiriou M., Mylonas I.G., Ninou E., Traka-Mavrona E. (2010). The Cultivation Revival of a Landrace: Pedigree and Analytical Breeding. Euphytica.

[50] Koutis K., Mavromatis A.G., Baxevanos D., Koutsika-Sotiriou M. (2012). Multienvironmental evaluation of wheat landraces by GGE Biplot Analysis for organic breeding. Agric. Sci..

[51] Smutná P., Mylonas I., Tokatlidis I.S. (2021). The Use of Stability Statistics to Analyze Genotype × Environments Interaction in Rainfed Wheat under Diverse Agroecosystems. Int. J. Plant Prod..

[52] Mylonas I., Sinapidou E., Remoundakis E., Sistanis I., Pankou C., Ninou E., Papadopoulos I., Papathanasiou F., Lithourgidis A., Gekas F. (2020). Improved Plant Yield Efficiency Alleviates the Erratic Optimum Density in Maize. Agron. J..

[53] Sinapidou E., Pankou C., Gekas F., Sistanis I., Tzantarmas C., Tokamani M., Mylonas I., Papadopoulos I., Kargiotidou A., Ninou E. (2020). Plant Yield Efficiency by Homeostasis as Selection Tool at Ultra-Low Density. A Comparative Study with Common Stability Measures in Maize. Agronomy.

[54] Papastylianou P., Vlachostergios D.N., Dordas C., Tigka E., Papakaloudis P., Kargiotidou A., Pratsinakis E., Koskosidis A., Pankou C., Kousta A. (2021). Genotype × Environment Interaction Analysis of Faba Bean (*Vicia faba* L.) for Biomass and Seed Yield across Different Environments. Sustainability.

[55] Bilsborrow P., Cooper J., Tétard-Jones C., Średnicka-Tober D., Barański M., Eyre M., Schmidt C., Shotton P., Volakakis N., Cakmak I. (2013). The effect of organic and conventional management on the yield and quality of wheat grown in a long-term field trial. Eur. J. Agron..

[56] Rempelos L., Wang J., Sufar E.K., Almuayrifi M.S.B., Knutt D., Leifert H., Leifert A., Wilkinson A., Shotton P., Hasanaliyeva G. (2023). Breeding Bread-Making Wheat Varieties for Organic Farming Systems: The Need to Target Productivity, Robustness, Resource Use Efficiency and Grain Quality Traits. Foods.

[57] Mikó P., Löschenberger F., Hiltbrunner J., Aebi R., Megyeri M., Kovács G., Molnár-Láng M., Vida G., Rakszegi M. (2014). Comparison of bread wheat varieties with different breeding origin under organic and low input management. Euphytica.

[58] Mitura K., Cacak-Pietrzak G., Feledyn-Szewczyk B., Szablewski T., Studnicki M. (2023). Yield and Grain Quality of Common Wheat (*Triticum aestivum* L.) Depending on the Different Farming Systems (Organic vs. Integrated vs. Conventional). Plants.

[59] Papadakis I. (1929). Greek types of wheat. Scientific Bulletin of the “Special Station of Plant Breeding in Thessaloniki”.

[60] Payne R.W. (2009). GenStat. WIREs Comput. Stat..

