# Supplementary material for: Bread Wheat Landraces Adaptability to Low-Input Agriculture
Source: Plants (Basel). 2023 Jul 6;12(13):2561. doi: 10.3390/plants12132561 (PMC10347258; doi:10.3390/plants12132561)
Supplement: Supplementary file 1 [file plants-12-02561-s001.zip › plants-2447935-supplementary.pdf]

Table S1. ANOVA and means of bread wheat landraces and cultivars in each of the four evaluation environments for HD, during the 2nd cultivation season (2014-2015).

2nd season	df	L2-AUTH	L2-IPGRB	C2-IPGRB	O2-IPGRB
Genotypes	7	**	**	**	**
Blocks	3	ns	ns	ns	ns
error	21				
CV%		0.6	2.0	0.4	0.7
**Genotypes**					
**-Landraces**					
X1		138.8a †	136.0a	135.8ab	136.8a
X2		139.3a	135.8a	135.5ab	136.0a
X3		136.5bc	134.8ab	134.5b	135.3ab
X4		134.3d	130.3c	131.0d	130.5c
-**Commercial Cultivars**					
X5		136.0cd	133.5b	132.8c	133.8b
X6		138.5ab	135.5a	136.5a	137.3a
X7		130.0e	125.0d	125.3e	124.8d
X8		130.8e	125.3d	126.0e	125.8d

F-probability values: ** p≤0.01; ns = not significant. †Means followed by the same letter in a column are not significantly different (Tuckey's test, *p*≤0.05).

Table S2. ANOVA and means of bread wheat landraces and cultivars in each of the four evaluation environments for PH, during the 2nd cultivation season (2014-2015).

2nd season	df	L2-AUTH	L2-IPGRB	C2-IPGRB	O2-IPGRB
Genotypes	7	**	**	**	**
Blocks	3	**	ns	**	*
error	21				
CV%		11.0	3.0	5.8	2.9
**Genotypes**					
**-Landraces**					
X1		117.0a †	115.0a	122.5a	129.3a
X2		114.5a	105.0ab	120.0a	123.3a
X3		121.3a	97.0abc	115.0a	126.3a
X4		90.0b	82.5bcd	89.3b	88.0b
-**Commercial Cultivars**					
X5		61.3c	68.8d	68.8c	71.3c
X6		62.0c	60.0d	63.8c	68.3c
X7		58.8c	62.5d	67.5c	70.0c
X8		67.5bc	78.8cd	87.5b	93.3b

F-probability values: * p≤0.05; ** p≤0.01; ns = not significant. †Means followed by the same letter in a column are not significantly different (Tuckey's test, *p*≤0.05).

Table S3. ANOVA and means of bread wheat landraces and cultivars in each of the four evaluation environments for EL, during the 2nd cultivation season (2014-2015).

**2nd season**	df	L2-AUTH	L2-IPGRB	C2-IPGRB	O2-IPGRB
Genotypes	7	**	**	**	**
Blocks	3	ns	ns	ns	ns
error	21				
CV%		5.5	2.0	4.2	7.9
**Genotypes**					
**-Landraces**					
X1		12.3a †	10.1ab	10.0bc	11.0abc
X2		13.1a	9.7abc	10.7ab	11.8ab
X3		12.5a	11.1a	11.5a	12.3a
X4		9.0bc	8.9bcd	9.0def	9.9bcde
-**Commercial Cultivars**					
X5		9.0bc	8.2d	8.6ef	8.4e
X6		8.6c	8.1d	8.2f	8.9de
X7		10.0b	9.9abc	9.9bcd	10.4abcd
X8		9.1bc	8.5cd	9.4cde	9.7cde

F-probability values: ** p≤0.01; ns = not significant. †Means followed by the same letter in a column are not significantly different (Tuckey's test, *p*≤0.05).

Table S4. ANOVA and means of bread wheat landraces and cultivars in each of the four evaluation environments for NS, during the 2nd cultivation season (2014-2015).

**2nd season**	df	L2-AUTH	L2-IPGRB	C2-IPGRB	O2-IPGRB
Genotypes	7	*	**	**	**
Blocks	3	ns	ns	ns	ns
error	21				
CV%		13.9	4.0	10.3	25.5
**Genotypes**					
**-Landraces**					
X1		32.8bcd †	29.8cd	30.7d	41.3abc
X2		33.4bcd	25.3d	26.9d	31.6bc
X3		30.9cd	26.2d	26.1d	29.5c
X4		56.9a	54.9a	51.0a	57.0ab
-**Commercial Cultivars**					
X5		43.5abc	43.1abc	50.1a	53.0abc
X6		56.4a	49.8ab	48.7ab	67.5a
X7		27.6d	31.4cd	31.3cd	41.8abc
X8		45.5ab	35.7bcd	40.4bc	40.4abc

F-probability values: * p≤0.05; ** p≤0.01; ns = not significant. †Means followed by the same letter in a column are not significantly different (Tuckey's test, *p*≤0.05).

Table S5. ANOVA and means of bread wheat landraces and cultivars in each of the four evaluation environments for TKW, during the 2nd cultivation season (2014-2015).

**2nd season**	df	L2-AUTH	L2-IPGRB	C2-IPGRB	O2-IPGRB
Genotypes	7	*	**	**	**
Blocks	3	*	ns	ns	ns
error	21				
CV%		13.2	8.0	8.0	10.7
**Genotypes**					
**-Landraces**					
X1		32.7ab †	35.7ab	42.1ab	35.3c
X2		32.7ab	40.4ab	45.3a	50.5a
X3		35.8ab	42.9a	42.2ab	46.2ab
X4		32.5ab	38.6ab	37.6bc	43.2abc
**-Commercial Cultivars**					
X5		28.0b	32.4b	33.2c	37.1bc
X6		31.9ab	31.9b	31.5c	34.5c
X7		34.4ab	37.1ab	47.3a	45.2abc
X8		40.3a	39.7ab	45.5a	46.1ab

F-probability values: * $p \leq 0.05$; ** $p \leq 0.01$; ns = not significant. †Means followed by the same letter in a column are not significantly different (Tuckey's test, $p \leq 0.05$).
